# ‘It Is Not My Business’: Adolescent Boys’ Experiences and Responses to Community Violence in Soweto, South Africa

**DOI:** 10.1007/s40653-026-00848-9

**Published:** 2026-03-17

**Authors:** Campion Zharima, Elton Mboneli, Lerato Tsotetsi, Stefanie Vermaak, Busisiwe Nkala-Dlamini, Avy Violari, Rachel Kidman, Amy Hammock, Janan Dietrich

**Affiliations:** 1 Faculty of Health Sciences, Perinatal HIV Research Unit (PHRU), University of the Witwatersrand, Johannesburg, South Africa; 2 Faculty of Health Sciences, African Social Sciences Unit of Research and Evaluation (ASSURE), University of the Witwatersrand, Johannesburg, South Africa; 3 Faculty of Humanities, Department of Social Work, University of the Witwatersrand, Johannesburg, South Africa; 4 Program in Public Health, State University of New York at Stony Brook, New York, USA; 5 Department of Family, Population and Preventive Medicine, State University of New York at Stony Brook, New York, USA; 6 School of Social Welfare, State University of New York at Stony Brook, New York, USA

**Keywords:** Community Violence, Adolescents, Male, Young people, HIV, Soweto, South Africa

## Abstract

Community violence is linked to adverse social and health outcomes such as trauma, depression and injury. South Africa has high rates of community violence, particularly in townships where higher rates of poverty and unemployment are reported. The purpose of this study was to understand adolescent boys’ experiences, perceptions, and responses to community violence.

We used a qualitative design, conducting in-depth interviews with 48 adolescent boys aged 15–19 in Soweto, South Africa. This sample was drawn from a larger longitudinal study of 498 young males, both living with and without HIV. Participants were asked about their experiences with community violence and related health behaviors. Interview transcripts were coded and analyzed using thematic analysis.

Results showed multiple forms of community violence including physical assaults, gender-based violence, gang-related attacks, robberies and vigilante or mob justice, with some incidents occurring within schools. Violence was perceived as routine and normalized aspects of daily life, often linked to drug or alcohol use, poverty and limited recreational opportunities. Violence was also connected to masculine identity, expressed through strength, retaliation, and gang affiliation as a show of loyalty and group defense. Coping strategies ranged from avoidance and emotional detachment to reliance on community policing forums or direct participation in vigilante justice. Some were skeptical toward relying on police because they did not consistently respond.

This research shows how community violence is embedded in the lived realities of adolescent boys. Interventions must address both current and historical causes by expanding socioeconomic opportunities, improving reforms in policing and school safety, and supporting gender-transformative mentorship for boys.

## Introduction

Community violence is a global public health concern, which is more prevalent in urban environments and inner-city contexts (McDonald et al., 2011). Its definition involves intentional acts of interpersonal violence occurring in public areas such as neighborhoods, schools and streets of communities, especially by individuals who are not related to a victim or conflict which had given rise to the violence (Centers for Disease Control and Prevention, 2025). This includes acts such as robberies, physical assaults, and gang-related conflicts (Dawson et al., 2023). Community violence is often shaped by multiple concurrent factors and may manifest differently in various sociocultural contexts. For instance, in the US, urban poverty and socioeconomic inequality are key determinants (Beardslee et al., 2021; Schuck & Widom, 2021), while in South Africa, the colonial legacy of apartheid, historical dispossession and systemic inequality continue to shape patterns of violence in low-income communities (Dinan et al., 2004).

While community violence can potentially affect any demographic, adolescents and emerging adults are especially vulnerable, often as both victims and perpetrators (Lowry et al., 1995; Warmanen et al., 2017; Ybarra & Petras, 2021). Exposure to community violence during this critical developmental stage has been shown to result in physical harm, psychological distress, and cognitive impairment (McCoy et al., 2015), and increased risk of engaging in criminal behavior (Petrich, 2024).

Extensive research has shed light on the prevalence and impact of community violence, particularly its link to mental health (Desmarais et al., 2014; Mootz et al., 2023; Mudhikwa et al., 2025). However, this research is predominantly quantitative, leaving a dearth of qualitative studies that are essential for understanding how adolescents perceive, cope with, and respond to violent contexts. Understanding the experiences of adolescent boys is useful for designing contextually relevant interventions that mitigate harm and promote their resilience.

## Literature Review

Violence remains a major public health concern, with the World Health Organization estimating that 1.25 million people die annually from violence-related injuries, and men being twice as likely as women to be killed (World Health Organization, 2024). In densely populated urban areas, community violence is often intertwined with systemic neglect, creating a ripple effect that perpetuates trauma, poverty and marginalization. Research in high risk urban settings in sub-Saharan Africa shows that nearly half of adolescents aged 15–17 report experiencing physical, sexual or emotional violence in a single year (Ezenwosu & Uzochukwu, 2025). Such exposure to violence affects the safety of young people and threatens their wellness by disrupting social cohesion in these communities. Moreover, structural factors such as poverty, unemployment, substance abuse and inequality may exacerbate these experiences (Peitzmeier et al., 2016), and can be linked to specific types of violence for adolescents in some studies (Sui et al., 2020).

Exploring the impact of community violence among adolescent males is critical, as young boys are more likely to witness and perpetrate violence (Forster et al., 2020; Rizzo et al., 2024), a dual exposure that embeds them in cycles of violence that may persist into adulthood (Forke et al., 2018; Moretti et al., 2006). Moreover, adolescence is a pivotal stage of development characterized by identity formation and social learning, during which they begin to reflect on who they are and their place in society (Branje et al., 2021; Crocetti, 2017). At this stage, exposure to violence can significantly affect psychological development and increase their vulnerability to mental health and behavioral disorders.

### Community Violence in South Africa

South Africa offers a critical context for examining community violence, as its rates of violence are among the highest in the world, including homicide, assault and robbery (Bowman et al., 2024; Seedat et al., 2009). High-density urban townships such as Soweto in Johannesburg and Khayelitsha in Cape Town are examples of communities particularly affected (Swartz & Scott, 2014). These settlements are also similarly marked by persistent challenges like unemployment, poverty and poor service delivery, which are all conditions that fuel violence (Seedat et al., 2009). Importantly, the origins of these communities lie in the legacy of apartheid-era spatial planning, where segregationist policies relegated non-white people to less affluent zones on the periphery of urban centers, reinforcing cycles of marginalization and structural violence (Christopher, 1989, 1990).

Thus, the history of violence in South Africa is rooted in apartheid, which institutionalized structural violence, police brutality and racially segregated development (Finchilescu & Tredoux, 2010). As a result, townships such as Soweto became sites of both state sponsored violence and resistance, with many cases of protests against poor service delivery to date (Wafer, 2012). Decades of historical exclusion and authoritarian rule have left an imprint on many communities across South Africa, contributing to widespread mistrust of law enforcement. Some scholars have argued that violence has been normalized in South Africa, and it has shaped the social fabric in lasting ways, influencing how young people experience violence today (Hoosen et al., 2022).

The historical backdrop of South African society is also useful in explaining why certain survival and coping strategies are adopted by adolescents. For instance, gang affiliation or substance abuse may emerge as adaptive responses to manage stress and trauma rather than mere deviant behaviors (McDaniel, 2012; Taylor et al., 2007). This is because for many adolescent males, violence can be rationalized as a means of survival, protection, or self-defense. Adolescent males are vulnerable to community violence as research shows its links to a range of adverse outcomes such as risky sexual behavior, substance abuse and low educational attainment (Fowler et al., 2009; Jagasia et al., 2024). These factors may further contribute to maladaptive coping mechanisms.

Many South African communities are shaped by overlapping social and health challenges including HIV, substance abuse and violence, which form part of the broader context of peri-urban and marginalized communities in which adolescents live (Hatcher et al., 2022). These intersecting challenges provide an important backdrop for understanding how community violence is experienced and normalized in such settings. South Africa bears one of the highest HIV burdens globally, with adolescents being part of a vulnerable demographic affected by the epidemic while also facing a heightened risk of mental health challenges (Inman et al., 2024; Milovanovic et al., 2021). The epidemic is deeply rooted in many communities, where internalized HIV stigma among adolescent boys has been linked to higher rates of depression, alcohol abuse and greater exposure to violence (Inman et al., 2024). Although this study does not examine HIV-related outcomes, this study includes both adolescents living with and without HIV to capture the broader social environments from which participants were drawn. Moreover, some research has shown that violence can impact youth access to health care, particularly sexual, reproductive and trauma care (Green et al., 2023).

The aim of this study is to explore how adolescent boys living in Soweto perceive, experience, and respond to community violence. It contributes to the growing body of evidence supporting the need for contextually grounded, youth-informed interventions that are rooted in historical awareness and community engagement. By centering the voices of adolescent boys and directly exploring community violence, this study gathers critical insights into how young people navigate violence in communities, not as victims or perpetrators only, but as active agents in their environments.

## Methods

This qualitative study draws on interviews with participants from the Tsamaisano study, a longitudinal cohort study conducted between 2020 and 2023 which explored the impact of violence on HIV transmission among adolescent boys and young men (Kidman et al. 2025a, b).

### Study Setting

This study was conducted at a health research facility located in Soweto, South Africa, serving the resident population in this geographical area. Soweto is a peri-urban area or township, located South-West of the city of Johannesburg, in the Gauteng province of South Africa, and has an estimated population of 1.9 million in an area of 63 km2 (South African History Online, n.d.). It was created in the 1930s as a result of segregationist laws (i.e., the Urban Areas Act in 1923) under South African apartheid, when black people were moved away from the inner-city areas (Davenport, 1970). Over time, it has grown into a densely populated area where residents continue to face significant challenges that limit their access to socioeconomic opportunities (Mathe, 2022).

At the same time, Soweto is also known for its strong sense of community cohesion, rich social networks, and long-standing traditions of collective action and resilience (Enaifoghe & Dlamini, 2021). The area has a well-established history of civic engagement, youth mobilization, and community-based organizations that play an important role in social support, health promotion, and violence prevention (Loren Kruger, 2017). In addition, Soweto hosts a relatively dense network of public health facilities, schools, and non-governmental organizations, which contribute to service accessibility and provide important points of engagement for adolescents and young people, some of which were leveraged in this study’s recruitment. These community strengths are important contextual factors for understanding how young men navigate life within this setting.

### Sampling and Recruitment

The parent study enrolled 498 males aged 15–19 years (251 living with perinatal HIV (PLHIV) and 247 HIV-negative) (Kidman et al. 2025a, b). PLHIV participants were recruited from local HIV clinics in the greater Soweto area and HIV-negative peers were recruited using community outreach in the same geographic areas. The HIV clinics served solely as recruitment sites for participants living with HIV. All study visits were conducted at a separate study-site clinic that is distinct from the HIV treatment clinics. Over a period of 12 months, all participants, regardless of HIV status, completed 3 in-clinic study visits (at enrollment, 6 months and 12 months) at the study site clinic and completed weekly mobile phone surveys over their enrolment period.

This paper reports on a sub-study in which 52 participants were further recruited from this pool for qualitative in-depth interviews using a criterion sampling approach. Participants were stratified based on IPV experiences (IPV experience and no IPV experience), HIV status (perinatally infected and uninfected), age groups (minors under 18 and adults 18 and older) and adverse childhood experiences (ACEs) (low, medium and high). These categories were formed to ensure equal representation of desired characteristics in the sample. This sampling strategy was designed to capture adolescents with diverse social and health experiences within a shared community and study context, enabling exploration of perceptions and experiences of community violence and intimate partner violence (IPV). Thus, participant HIV status was used to ensure sampling diversity rather than to inform the analytic focus of this paper.

### Study Procedures

The in-depth interviews were conducted by four local research assistants who had experience with in-depth interviewing and were proficient in the local languages spoken by participants. One of four research assistants (two men and two women) conducted each in-depth interview in the local language(s) preferred by participants (English, Zulu or Sotho). Using both female and male interviewers across the study was intended to minimize systemic bias related to interviewer gender. Research team members received standardized training in qualitative interviewing. The interviews were piloted, after which a senior researcher reviewed and provided feedback on how to improve the interviews as well as to refine the interview guide where necessary.

### Data Collection

A semi-structured interview guide was used to conduct the in-depth interviews, with topics including experiences of IPV in relationships, household violence, childhood abuse and community violence, sexual behaviors and, for those living with HIV, medication adherence, disclosure, and HIV management. Appropriate probes for each section were included to help guide the discussion and ensure that the relevant content is covered. This paper focuses on the themes of community violence, specifically the types of violence experienced in the community and participants’ reports on why and how it occurs. All interviews were conducted in private rooms located at the site, and all audio-recorded interviews ranging between 32 and 95 min in length. Regular weekly meetings were held to discuss emerging themes.

### Data Analysis Approach

All audio-recordings were transcribed verbatim, and those conducted in local languages were translated into English. A multi-disciplinary research team was involved in the analysis process, including South Africa based researchers with contextual and linguistic familiarity with the study setting, as well as senior researchers based outside South Africa. This composition enabled analytic reflexivity and ensured that interpretations were grounded in local social and cultural contexts while also including external perspectives.

We familiarized ourselves with the data by independently reviewing a set of transcripts and then meeting to discuss the recurring issues. This was followed by research assistants reading through the transcripts and compiling a coding framework with input from other research team members. As more transcripts were coded, the research team met several times to refine existing codes and add new codes and to further refine the codebook. Following the iterative refinement, the finalized codebook was systematically applied to all transcripts within NVivo, a qualitative data analysis software, where all responses related to community violence were identified, and relevant quotes were extracted for analysis. All text segments coded under community violence related parent and subcodes were retrieved, reviewed, and compared across transcripts to identify patterns, similarities, and differences in participants’ experiences and interpretations of community violence.

The research team engaged in an iterative thematic analysis process, involving repeated reading of coded data, comparison across participants, and the development and refinement of themes through team discussions. Analytic memos were used to document emerging interpretations, relationships between themes, and reflections on how participants understood and responded to community violence. These interpretive steps supported the development of higher-order themes that captured both shared and divergent experiences among participants.

In our analysis, participant sampling characteristics, including age group, HIV status, IPV experience, and ACEs category, were considered to support reflexive interpretation of the data. While our analysis did not involve a formal subgroup comparison, these characteristics were used to contextualize participants’ accounts and to explore whether particular themes appeared to vary across different social and life-course positions. This approach helped situate findings within participants’ broader lived realities while maintaining a thematic focus aligned with the study aims.

## Results

Of the 52 interviews, four were excluded from these analyses: one because of a consent inconsistency and three because of a lack of audio recording. Below, we present the findings from the remaining 48 in-depth interviews with adolescent boys living in Soweto, South Africa. Our analysis highlights their direct and indirect experiences with community violence, including how they perceive, cope with, and respond to violence in their lives. As such, we identified and report on four major themes which include: (1) types of community violence encountered, (2) perceptions and normalization of community violence, (3) coping strategies and responses to violence and, (4) the impact of violence on masculinity.

1. Types of community violence encountered

Participants reported experiencing or witnessing multiple forms of violence in their communities, including physical assaults in the form of vigilante justice, robberies, gang related attacks and sexual violence. Those who experienced physical violence and assault mentioned beatings, stabbings and shootings as incidents that occurred in public settings such as streets:

“*Like when we are sitting*,* you just notice people bottle fighting with each other. Also*,* at the dice game*,* you notice one just getting aggressive…and a gunshot goes off just now. Now in my townships*,* they are using a lot of blank guns. They just shoot you now. And they know it won’t fatally hurt you*.” — (IDI_36, 21 years, HIV-Negative).

In this case, participants reported a common form of this violence as mob justice, where community members would assault individuals who were suspected of or caught committing crimes, often bypassing the involvement of law enforcement:

“*Every day*,* they steal. When you steal*,* we find you*,* we beat you.*” — (IDI_32, 19 years, HIV-Negative).

Some participants also shared how violence takes place very frequently:

“*Yeah*,* it doesn’t go a day without a person being beaten [publicly]*,* but sometimes they take them into the yards and beat them there.*” — (IDI_38, 20 years, PHLIV).

Some participants pointed out how some of the violence often takes place in contexts that involve alcohol, in some cases targeting women in public spaces:

“*In the townships there are a lot of bottle stores and taverns. So*,* when people are angry*,* there is actually going to be a fight. If they are drunk*,* sometimes a small thing will spark a fight.*” — (IDI_35, 21 years, HIV-Negative).

“*I think some time last month*,* I saw a man abusing some women in the street. The man was drunk. She even opened a case*,* and the guy was arrested.*” — (IDI_50, 18 years, HIV-Negative).

Robberies and muggings were mentioned as occurring frequently. Participants reported incidents of street muggings, phone snatching and even car hijackings, often through violent means, usually done to sustain the perpetrator’s drug use.

“*There are guys that mug people*,* I have seen them they appear in corners at night most of them are guys that take drugs*,* so they mug people to buy drugs… they stabbed this other guy next to a clinic*,* where most people go to connect Wi-Fi or to the Internet*,* he was standing there holding his phone and they appeared*,* stabbed him*,* and took his phone and ran away.*” – (IDI_28, 16 years, HIV-Negative).

These quotes show how violence in this community can be tied to opportunism, where perpetrators take advantage of situations or people, and/or become desperate. Another form of violence commonly mentioned was gang related violence, with participants naming various local gangs and describing turf wars and retaliatory violence:

“*With violence in my community*,* I can say there are gangs…because people form gangs*,* it is not easy having to walk at night*,* when you walk at night in the ghetto*,* you can get stabbed or mugged.*” – (IDI_42, 18 years, HIV-Negative).

“*Yes*,* I’m in a gang. So*,* if I walk alone*,* one-one*,* they’ll catch me. You know*,* I’m not safe due to the community violence. I see it when they beat someone…I have enemies*,* I have friends*,* I have everything*” – (IDI_32, 19 years, HIV-Negative).

Finally, participants also shared how different forms of violence, including fights, stabbings and group attacks were experienced at school, often with little intervention from school authorities. One participant described his experience of being attacked on school premises:

“*When they chased me*,* they caught me and they kicked me. All of them. After kicking me*,* they went to my friends’ class and when they found him*,* they took a knife and then they stabbed him on the chest twice. Luckily*,* the knife didn’t enter.*” – (IDI_16, 19 years, PLHIV).

Some reported how school-teachers refrain from intervening in cases where school violence occurs and how this often leads to calling police for help:

“*Obviously*,* the police must come and assist because the teachers don’t want to get involved. They don’t assist because they are also scared because they also get beaten up when they try and solve problems that have nothing to do with them. When this issue goes outside the school yard there’s nothing that the teachers can do*.” – (IDI_23, 21 years, PLHIV).

This indicates that schools are sites of violence and that the violence that takes place there also mirrors the dangers in the broader community.

2. Perceptions of community violence and its normalization.

Given the reported pervasiveness of violence in this community, many participants described it as routine and said that they have accepted it as part of their everyday lives:

“*In the township*,* I feel like physical violence is something that happens every day*,* everywhere because as human beings*,* we’re not the same. Some have stress*,* some of them*,* it’s beyond their control*,* some of them don’t even know what’s happening with them*,* but then yeah*,* physical violence has become a way of life.*” – (IDI_44, 21 years, HIV-Negative).

“*I’m used to this thing of seeing [violence]*,* because even in the township there were some taxi drivers shooting stories. I have seen a lot I don’t want to lie. So I’m used to these things.*” – (IDI_30, 17 years, HIV-Negative).

As such, participants felt that violence was so routine that they were now used to it and were no longer shocked by it, as some had become emotionally detached:

“*I saw someone being beaten. Some of us we don’t mind because its none of our business*,* not that we need to call the police*,* we will only talk about seeing him being beaten.*” – (IDI_06, 20 years, PLHIV).

This illustrates how repeated exposure to violence rendered it mundane and had shaped adolescents’ perceptions of what was expected or ordinary in their environments.

For others, violence was viewed as a product of broader structural issues, such as drug abuse and lack of recreational spaces for young people:

“*There is a lot of violence in the hood. Like things that happen in the hood are the ones that make people become addicted to drugs…I think maybe it’s because of the situation in the hood that makes people turn out to be the way they are*,* I think maybe it’s because the hood doesn’t have parks or places that people can go and relax*,* chill*,* or become active in.*” – (IDI_49, 20 years, HIV-Negative).

A few participants attempted to morally justify violence, especially when it was framed or used as a means of supporting dependants or corrective action or punishment for perceived wrongdoing as the following two share:

“*Yes. For me to be able to feed my child…. Anything to do with money… I do that thing.*” – (IDI_32, 19 years, HIV-Negative).

“*If you are too forward*,* my brother*,* and you are taking someone’s girlfriend*,* then they come and kick you? We laugh and come and ask for peace after.*” – (IDI_04, 17 years, PLHIV).

Similarly, another participant shared the view that children’s misbehaviour stems from a lack of discipline, which he attributes to parents no longer using violence as physical punishment:

“*Parents don’t groom or warn their kids from doing bad things. They just let them be. I think it’s also because they stop beating these kids up to make them go back to normal kids.*” **–** (IDI_49, 20 years, HIV-Negative).

This sentiment reflects a view that corporal punishment is a necessary form of punishment rather than a form of violence.

3. Coping strategies and responses to violence.

Participants described several strategies for coping with and responding to violence in their communities. These strategies include both avoidant and active ways of adapting to the environment, with some disassociating themselves from violence while others engage in violence for protection and survival.

For most participants, avoiding conflict and violent spaces was the most common strategy, as many emphasized the importance of not getting involved in or reacting to other people’s issues:

“*When I hear people arguing*,* I don’t want to listen to their argument*,* I just leave. Just like that. If they hurt each other*,* it is not my business*,* that’s the way I am. Even if they kill each other*,* I don’t want to involve myself.*” – (IDI_40, 21 years, PLHIV).

*“I think it’s best for you to move and try to avoid.”* – (IDI_02, 16 years, HIV-Negative).

Avoidance in this case seems to be rooted in the belief that involvement may invite retaliation, which puts one at risk for physical harm. Some participants resorted to emotional detachment, a coping strategy to manage psychological trauma when confronted with violence, as shared by the following:

“*I once witnessed something. It was someone who stabbed another one over a cement*,* but it was not happening in my own community…I felt nothing.*” – (IDI_41, 18 years, PLHIV).

“*It is normal. If someone is fighting over there*,* I wouldn’t say it is affecting me. Like people taking out pangas [knives] to stab each other. It happened one day when we just finished smoking our pipe and around the corner*,* we just witnessed a guy beating another one on the head with a bottle.*” – (IDI_36, 21 years, HIV-Negative).

In some cases, participants also shared a deep scepticism toward law enforcement and justice systems, describing these as ineffective or absent. As a result, some participants and the broader community who shared this sentiment often resort to their own ways of solving their problems, including engaging in community led vigilante action:

“*If someone did something wrong*,* the community beat them and kill them…and sometimes I also get involved when they beat someone.*” — (IDI_47, 18 years, PLHIV).

*“On this side*,* we don’t call the police. Because even if they call the police*,* it’s the same. They don’t help with anything.”* — (IDI_29, 18 years, HIV-Negative).

In one specific case, a participant explains how they intervened to avenge the rape of a girl from their community, deliberately bypassing the police:

“*It never got to a point where SAPS [South African Police Services] got involved*,* but us as youth*,* I feel like at that time*,* we’re the only ones who took charge of it. We got into a fight because the girl was from the same hood as most of us. We ended up fighting with the person who raped the girl*,* that’s how it ended.*” — (IDI_44, 21 years, HIV-Negative).

Participants further highlighted how some communities have, in response to the violence, relied on community policing forums (CPFs). These often also use violence and do not work with the police:

“*In the community I stay*,* there are patroller CPFs. When they find someone*,* they beat that person even without hearing what that person is trying to say…I won’t say they are working with the police because the police avoid them in most cases.*” — (IDI_19, 18 years, HIV-Negative).

This reflects a broader trend, where communities have resorted to informal systems in the absence of trusted authorities for crime fighting. Thus, coping with violence involves a mix of strategies that reflect both resilience and vulnerability.

4. Impact of community violence on masculinity.

For many adolescent boys, community violence was not only a lived reality but a force that affected and shaped how they understood themselves and their masculinity in these settings. In some cases, traditional forms of masculinity, with ideals such as physical and emotional strength as well as dominance, were reinforced through these experiences. This was shown in cases where participants were both victims and perpetrators of violence. Thus, the pressure to demonstrate strength and defend oneself from violence often pushed young men into violent situations, even when they might have preferred to avoid conflict. One participant explained how this has led to tragic consequences for another young man:

“*Some boy in my community…he was being bullied at school and when he reports at home*,* they never helped him. They told him that he is a boy he should fight for himself*,* and he couldn’t and ended up hanging himself…even at school teachers couldn’t assist him.*” — (IDI_19, 18 years, HIV-Negative).

Across different interviews, participants expressed how young boys are expected to fight their own battles without support, by showing strength, defending themselves and not backing down from violence. Retaliation and revenge were common ways of showing strength; young men described these as quests to restore their sense of justice and to signal toughness. One participant describes his experience getting revenge from a robber:

“*We were about to turn on another corner [when] they [robbers] took out a knife. They took the phone and the money. We manage to run away me and my brother…We went after them*,* we did find them and we beat them so that they will never do what they did to us.*” — (IDI_51, 17 years, PLHIV).

In other cases, masculinity and violence can intersect with gender dynamics, as in one case where a participant describes being publicly beaten by the family of a woman he had verbally confronted, after pulling a knife on her as a means to ‘silence her’ during a conflict:

“*I was once arrested for nothing. Like this lesbian…this lesbian started messing with me*,* you know. They fought me and I took out a knife in front of their family. The whole family came out and beat me. I was beat by the community. We were talking she came near me because my words were more powerful than hers cause she’s a woman. I never laid a hand on her. I took out the knife to make her quiet. She ran at home and said I almost stabbed her.*” — (IDI_32, 19 years, HIV-Negative).

This illustrates how the participant perceived the confrontation as an attack on his manhood and felt it necessary to respond with the threat of violence.

In some cases, violence was described as a requirement for acceptance into certain groups or gangs. Some participants spoke about how they were expected to commit acts of violence or violent crimes, such as stealing and stabbing, to prove their loyalty or toughness to the group:

“*For you to be in the group you have to show that you are capable of such thinks so like yes in the event we took hats and what*,* what from people but I have never stabbed anyone.*” — (IDI_30, 17 years, HIV-Negative).

“*To gain respect*,* you have to beat the other members of another group…they have to fight for no reason so that it can be known that this one is a fighter not to play with. [For gang initiation] Some say you have to steal something or you have to stab someone before you become a member.*” — (IDI_25, 18 years, HIV-Negative).

These experiences demonstrate how violence may not always be an expression of personal grievances but also as performative masculinity, or rites of passage which boys go through to earn their acceptance, identities and status within social groups.

For some participants, violence for young men was a way to gain respect and assert their masculinity in order to navigate threatening environments. One participant explained how his affiliation with a gang in his community changed his situation, giving him power and security:

“*I became more. I lived with people who are feared*,* you know. No one could do anything to me now. I support my child. Let me say…the money that I’m making now*,* for my child*,* it’s enough money.*” — (IDI_32, 19 years, HIV-Negative).

In this case, aligning with violence was more than just for safety: it intersected with his sense of empowerment to facilitate his responsibility to provide for his child. This suggests that his perceived role as a father to provide for his child was tied to his affiliation with a gang that committed violent crimes in order to gain resources.

In addition, the desire for dominance and recognition was highlighted as one of the reasons why participants experienced peer and gang violence. More specifically, participants described how jealousy, competition and the need to maintain their status could escalate into violent conflicts as shared by the following two participants:

*“Sometimes I think of maybe its jealousy*,* when someone has something you don’t and you want to see yourself on top of him*,* you beat him*,* others bully you.”* — (IDI_03, 17 years, PLHIV).

*“Sometimes like there is an event*,* let’s say me and you we are dancing…Like I defeat you in dancing and the crowd is going with me*,* you get jealous and you end up wanting to stab me.”* — (IDI_30, 17 years, HIV-Negative).

In both accounts, public humiliation or being overshadowed by a peer is described as provoking and often met with violent responses. One participant goes further to express his belief in the gang as a source of protection, and their shared masculine responsibility to protect each other as gang members:

“*It’s the law that the blood of a brother cannot be dripping down. I cannot be beaten while my gang member is here.”* – (IDI_29, 18 years, HIV-Negative).

The previous participant highlights the symbolic and emotional function of gangs as spaces of loyalty, belonging and protection as the quote reinforces the idea of collective strength and deterrence that comes from gang membership.

## Discussion

Our findings illustrate the multiple ways in which community violence is embedded in the lives of adolescent boys in Soweto, shaping their perceptions, coping or responses and how it affects their masculinity. As a result, we report on four key themes: (1) the normalization and desensitization to violence, (2) the adoption of vigilante justice in the absence of effective policing, (3) how violence frequently occurs and extends into shared spaces like schools, and (4) the intricate link between violence and masculinity in young men. These themes shed more light on the context in which adolescent boys and young men are growing up and highlights some of the urgent areas for intervention. In this section, we further discuss each of these findings and explore their implications for intervention efforts.

Many participants reported violence as a routine feature of daily life, reflecting a normalization of community violence. This perception can stem from repeated exposure, which research in South African communities (Seedat et al., 2009) and among African American communities in the United States has shown can lead to pathological adaptation through desensitization (Bacchini & Esposito, 2020; Gaylord-Harden et al., 2016). In our study, desensitization appeared to function as an adaptation strategy as participants choose not to intervene when witnessing violence and presented themselves as indifferent or unaffected. Normalization in this case reflects the commonality of violence in their environment, while desensitization represents a coping mechanism to manage the constant threat posed by violence.

Moreover, adolescents in our study shared a range of ways they coped with and responded to community violence. While global literature often identifies avoidance as the most common approach (Fenner et al., 2024; Gaylord-Harden et al., 2016; Teitelman et al., 2010), our findings also highlighted emotional detachment and, for some, gang affiliation as prominent strategies. Another study documented religion and spirituality as key coping mechanisms, such as among Latino adolescents in the US (Epstein-Ngo et al., 2013), but these were not observed in our study. This may be a reflection of differences in cultural norms or available support systems for young men in our study.

Several scholars have proposed frameworks for understanding coping mechanisms. For instance, some scholars group coping mechanisms into three broad categories: getting through (enduring), getting away (avoiding) and getting along (adapting) (So et al., 2021). Others distinguish between emotion-focused coping, which manages the distress associated with violence, and problem-focused coping, which seeks to address its source (Baker & Berenbaum, 2007; Lazarus & Folkman, 1984). Applying these frameworks to our findings, participants’ strategies such as emotional detachment and avoidance align with both getting along and getting away approaches. This is because they both emphasize managing emotions rather than directly confronting or changing the source of stress, which is the core of emotion-focused coping. Strategies such as gang affiliation can be seen as a response to a complex interplay of economic, social and psychological factors, including historical marginalization (Baird et al., 2022; Lauger & Lee, 2020; Vigil, 2003). By situating these actions within coping theory, we gain a clearer lens to interpret them as intentional emotion-focused strategies, which highlights opportunities for supporting healthier, more constructive coping pathways for young men. However, reliance on emotion-focused strategies can offer short term relief but eventually lead to maladaptive practices such as depression, substance abuse or self-harm (Boxer & Sloan-Power, 2013).

In our study, most participants coped with community violence through emotional detachment or avoidance, while some actively participated in violence to mitigate their vulnerability. One prominent form of active engagement described by participants is vigilante action, including ‘mob-justice’ and gang membership These practices often stem from participants’ distrust in formal authorities such as law enforcement and school authorities, a pattern also reported in literature (Bruch & Soss, 2018; O’Brien & Tyler, 2019). In other studies, this distrust has been linked to perceptions of police ineffectiveness or failures in the criminal justice system (Shodunke et al., 2023). In our context, participants described mob justice as a paradoxical response, while violence was condemned in principle, it was simultaneously viewed as the most accessible form of bringing accountability and punishment for crimes in communities. This reflects broader debates in the literature on how weakened trust in law enforcement fuels reliance on informal, and often violent, mechanisms of justice (O’Brien & Tyler, 2019). Literature reports that rebuilding trust requires procedural justice approaches (Melkamu & Teshome, 2023), which in South Africa could include consistent, visible, and fair policing practices, investment in community-based safety initiatives (Dreier & Lake, 2019; Lamb, 2021).

Moreover, participants reported that community violence often extends into schools, where group conflicts spill over and continued with minimal protection or intervention from school authorities. This lack of safety undermines students’ sense of security in environments typically viewed as safe havens. Research supports this dynamic, showing that negative experiences in school, such as being marginalized or unfairly treated, can diminish trust in authorities later in life (Bruch & Soss, 2018). These findings highlight the need for formal reforms in both policing and school safety protocols that are reflect the lived realities of affected communities and actively foster trust. An American community-based participatory action research project involving adolescents and parents used two concurrent programs, an 8-week online course and a youth summit, to address violence (Oscós-Sánchez et al., 2021). The Violence Prevention Program reduced violence outside school after 12 months, while the Positive Youth Development Program reduced in-school violence after 6 months.

Finally, participants’ narratives showed a link between violence and notions of masculinity. Literature on hegemonic masculinity shows that manhood is often associated with traits such as strength, endurance and dominance, qualities which validate masculine identities (Beasley, 2008; Connell & Messerschmidt, 2005). However, these rigid masculine expectations can leave young boys isolated and unsupported, particularly in contexts where exposure to violence is frequent and survival demands constant performance of traditional masculine norms. Our findings further elaborate on this dynamic, showing that gang membership provides a means for participants to construct masculinities grounded in show of strength, solidarity and dominance. As such, gangs become not just protective networks but pathways for masculinity to be enacted and affirmed (Deuchar, 2018). These dynamics reflect an environment where violence is normalized and instrumental to resolving conflict and asserting status.

As noted in the above discussion, our findings point to clear priorities for intervention and prevention. The prevalence of violence in schools suggests that improving school safety systems is urgent as schools are extensions of the broader community (Oscós-Sánchez et al., 2021). Moreover, the widespread use of vigilante justice as a result of the distrust in law enforcement highlights the need for reforms that make law enforcement more visible and responsive in communities (O’Brien & Tyler, 2019). Addressing the link between violence and masculinity requires interventions that include gender transformative approaches for young men, including mentorship programs where masculinity is reconfigured to support boys towards exploring alternative masculine roles and healthier coping (Dworkin et al., 2013; Gibbs et al., 2020). For gang membership, interventions need to combine psychosocial support with economic opportunities, including job creation schemes, structured after school programs, which have been successfully demonstrated in other high violence settings (Baird et al., 2022). Together, these multi-layered strategies can simultaneously address weak institutions, social norms and structural vulnerabilities that perpetuate cycles of community violence.

### Strengths and Limitations

This paper uses a qualitative approach to complement and deepen the insights provided by existing quantitative research on this topic. By focusing on the experiences of adolescent boys, it offers a unique perspective on how young people transition into adulthood and how their environments shape their development. The inclusion of adolescents living with perinatally acquired HIV (PLHIV), alongside HIV-negative peers, enhances the depth of analysis by capturing diverse lived experiences within a shared community context. This diversity strengthens the study’s transferability to similar settings where adolescents navigate overlapping structural vulnerabilities as in the context of Soweto.

However, our study’s findings may not be widely generalizable beyond Soweto, the specific context of the study. In addition, interviews were conducted by multiple researchers (male and female), which although potentially enriching the data, may have brought variations in the way interviews were conducted, including how questions were asked or interpreted. Moreover, participants’ responses may have been affected by social desirability bias: while efforts were made to ensure confidentiality and build rapport, participants may still have tailored their responses to align with perceived expectations.

## Conclusion

Our study explores the lived experiences of adolescent boys in relation to community violence, shedding light on its complex and context-specific nature within the setting of Soweto. Their narratives reveal exposure to various forms of violence, ranging from physical assaults and gang conflict to vigilante violence, including how they perceive, rationalize and respond to these threats. Drawing on these insights, responsive strategies for interventions must address both individual and structural level drivers of community violence. Such interventions should include expanding socioeconomic opportunities to reduce the appeal of gangs, strengthening trust in formal accountability systems through reforms in policing and school safety and gender transformative programs to redefine masculinity in non-violent and constructive ways.

## Data Availability

No datasets were generated or analysed during the current study.
